# Differential Abundance of Brain Mitochondrial Proteins in Yak and Cattle: A Proteomics-Based Study

**DOI:** 10.3389/fvets.2021.663031

**Published:** 2021-08-31

**Authors:** Xiaoming Ma, Qiang Zhang, Yongfu La, Donghai Fu, Hiu Jiang, Pengjia Bao, Xiaoyun Wu, Min Chu, Xian Guo, Ping Yan, Chunnian Liang

**Affiliations:** ^1^Animal Science Department, Lanzhou Institute of Husbandry and Pharmaceutical Sciences, Chinese Academy of Agricultural Sciences, Lanzhou, China; ^2^Key Laboratory for Yak Genetics, Breeding, and Reproduction Engineering of Gansu Province, Lanzhou, China; ^3^Institute of Animal Husbandry and Veterinary Medicine, Tibet Academy of Agriculture and Animal Sciences, Lhasa, China; ^4^State Key Laboratory of Hulless Barley and Yak Germplasm Resources and Genetic Improvement, Lhasa, China

**Keywords:** yak (*Bos grunniens*), mitochondria, proteomics, plateau adaptability, iTRAQ

## Abstract

The plateau adaptability and stress resistance of yaks are widely known based on their capacity to survive under severe habitat conditions. However, a few studies on brain mitochondria have characterized these adaptations at the protein level. We identified and quantified the brain mitochondrial proteins using isobaric tags for relative and absolute quantification (iTRAQ) and Proteomics. Western blotting was used to verify changes in the expression of target proteins. A total of 57 differentially abundant proteins (DAPs) were identified in the yak brain tissue. Gene Ontology (GO) analysis showed molecular functions of these DAPs including downregulated oxidoreductase activity but upregulated coenzyme binding. Significantly enriched biological processes were oxidation–reduction process (downregulated) and small molecule metabolic processes (upregulated). STRING protein interaction analysis indicated a complex interaction between dehydrogenase, transaminase, and ATP synthetase families. Reactome pathway analysis highlighted that the majority of DAPs participated in aerobic metabolic pathways such as metabolism, citric acid cycle, and respiratory electron transport. Immunoblotting confirmed that changes in FKBP4 and ATPAF2 expression were consistent with the results of mass spectrometry. We performed a high-throughput screening to identify DAPs in brain mitochondria between yak and cattle, which could explain the plateau adaptability of yaks.

## Introduction

Yak (*Bos grunniens*) is a unique and economically important animal on the Qinghai-Tibetan Plateau (QTP) (1). Yaks are distributed in areas with low temperatures (average annual value ≤0°C), big day–night temperature difference (above 15°C), short growing season of herbages (110–135 d), high radiation intensity (annual radiation amount of 140–195 KJ/cm^2^), and low oxygen partial pressure (below 110 mm Hg) (2). Due to the special ecological environment and strong natural selection, yaks have developed strong adaptability to natural environment conditions such as extreme cold, low oxygen, strong ultraviolet light, and nutrition stress in cold season (3). In contrast, domestic cattle (*Bos taurus*) are raised in agricultural areas at low altitudes, with abundant feeding sources, where they can grow throughout the year (4). In response to the harsh natural environment of the QTP, yaks have developed adaptive traits for long-term storage of nutrients and for managing hypoxia, including structural changes in internal organs and highly efficient oxygen transport (5).

The central nervous system of vertebrates is highly sensitive to hypoxia. As a long-term response to hypoxia, yaks possess special adaptive mechanisms. Even in the resting state, the brain requires high energy consumption and basic metabolism to maintain its normal functions (6). Studies have shown that the brain is highly sensitive to environmental oxygen. Although the brain only accounts for 2% of the total body weight, healthy brain tissue can obtain about 15% of the total cardiac output, and its oxygen consumption is as high as 20% of the total oxygen consumption of the body (7). How such a high oxygen utilization rate is achieved in the nervous system has remained a mystery. The oxygen delivered to brain tissue plays a crucial role in electrophysiological activities such as oxidative phosphorylation pathway, continuous ATP synthesis, and maintenance of sustainable energy supply (8, 9). Previous studies have reported that the unique brain morphology of yaks can adapt to high altitude hypoxia (10, 11). In addition, an analysis of yak mitochondrial genes revealed their associations with mitochondrial and oxidative phosphorylation, suggesting that the yak energy metabolism-related genes may change under natural pressure (12, 13).

Despite many studies on the plateau adaptability of yak, only a few have investigated the proteomic basis of this adaptation, including how modifications in aerobic respiration may contribute to efficient energy utilization. Being the site for the final oxidation of sugars, fats, and amino acids to release energy, mitochondria are the key organelles for oxidative metabolism in eukaryotic cells (14). Mitochondria also function to regulate the cell cycle and cell growth and participate in cell differentiation, cell signaling, and apoptosis (15). Characterization of the mitochondrial proteome of yaks may help to understand the molecular basis of their environmental adaptability.

Yaks and cattle have a close evolutionary relationship; they belong to the same genus. Despite differences in their environmental habitats and physiology, there are similarities in their metabolic activities. Therefore, yak is considered as an appropriate model to explore the molecular mechanisms underlying plateau adaptations. We performed differential proteomic analysis using mitochondria extracted from the brain tissue of yaks and cattle. We identified differentially expressed proteins (DAPs) within the mitochondrial aerobic respiration pathway using isobaric tags for relative and absolute quantitation (iTRAQ) technology. We conducted a comparative proteomic analysis of yak and cattle brain mitochondrial proteins, identified DAPs and their related functions and pathways, and constructed protein–protein interaction networks. Based on our findings, we present a genetic mechanism for low-oxygen adaptation and provide new clues for understanding brain mitochondrial function.

## Materials and Methods

### Specimen Collection and Storage

All yaks and cattle were handled in strict accordance with good animal practices that complied with the Animal Ethics Procedures and Guidelines of the People's Republic of China. The study was approved by the Animal Administration and Ethics Committee of Lanzhou Institute of Husbandry and Pharmaceutical Sciences of the Chinese Academy of Agricultural Sciences (Permit No. 2019-002).

**Figure 1:**
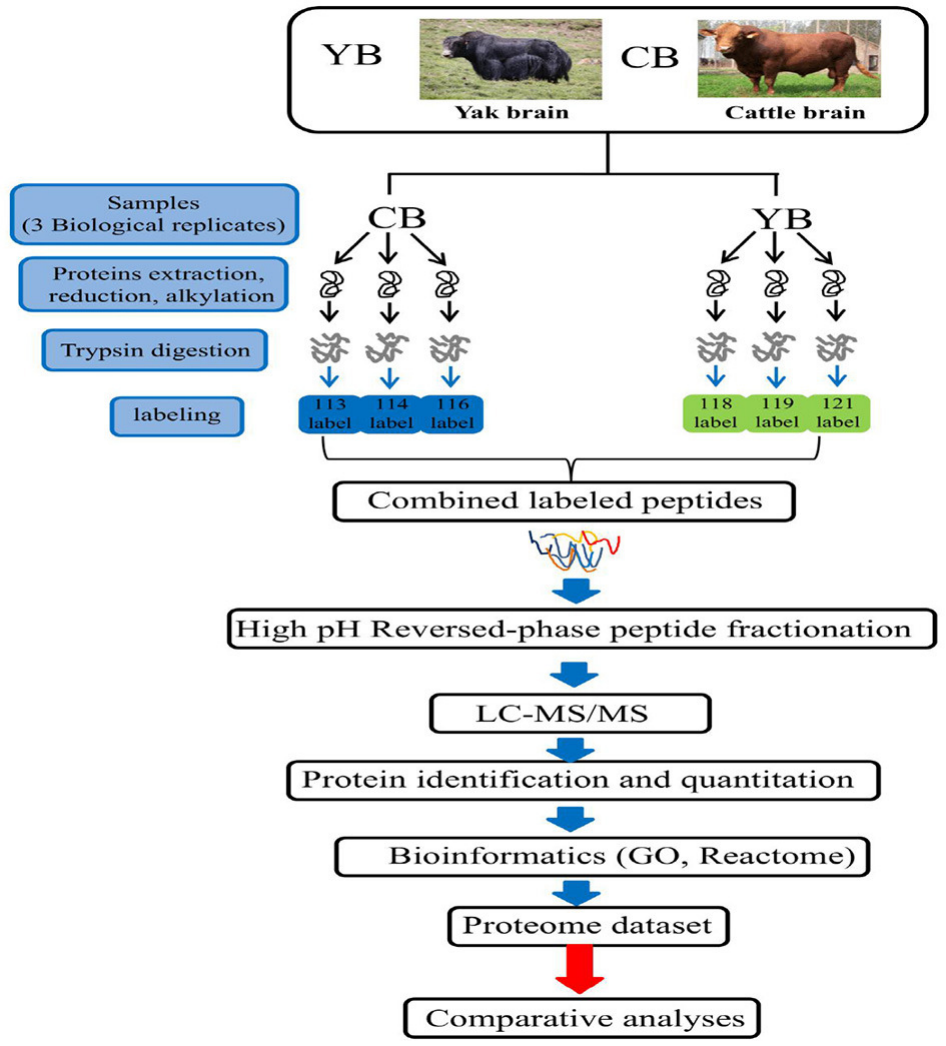
The iTRAQ LC-MS/MS strategy for quantitative proteomic analysis of brain mitochondrial proteins in yak and cattle.

The yaks used in this study were obtained from the grazing area of the Ashidan Mountain region of Qinghai Province (altitude of 2,720 m), which is located in the Datong County, Qinghai Province. The Datong Breeding Farm in the Qinghai Province is located between a latitude of 37° 11′-37° 32′ north and a longitude of 100° 52′-101° 26′ east in the southern foothills of Daji Mountain in the Qilian Mountain range. Cattle tissues used in this experiment were obtained from three healthy adult female Angus cattle. All cattle were from Angus Cattle Farm, Wuwei, Gansu Province at an altitude of 1,350 m. The yak and cattle investigated in this study were free grazing. The yak brain tissue was obtained from 3.5-year-old polled female yaks (*n* = 3). The cattle brain tissue was obtained from 3.5-year-old female cattle (*n* = 3). After animal sacrifice, the whole fresh brain from each animal was collected and washed with ice-cold phosphate-buffered saline (PBS). Brain tissues were cut in to small pieces, pulverized, mixed in liquid nitrogen, and stored at −80°C until use. Three individuals of yaks were named 113 (yak-1), 114 (yak-2), and 116 (yak-3), while three individuals of cattle were named 118 (cattle-1), 119 (cattle-2), and 121 (cattle-3) (Figure 1).

### Mitochondrial Isolation and Protein Preparation

Mitochondria were isolated using the ProteinExt Mammalian Mitochondria Isolation Kit for Tissue (Transgen Biotech, #DE501). The mitochondria obtained were assessed for their content and purity using 0.5% Janus Green B staining and light microscopy. Total protein was extracted from isolated mitochondria using the Protein Extraction Kit (Solarbio, #BC3710-50T). After removal of highly abundant proteins by three elutions, all protein samples were quantified using the bicinchoninic acid (BCA) method (Enhanced BCA Protein Assay Kit; Beyotime, #P0010S).

### Proteolysis and Peptide Labeling

Total protein (200 μg) per sample was adjusted to an equal volume (100 μL) with 8 mol/L urea buffer. DL-dithiothreitol was added to each sample at a final concentration of 10 mmol/L and the samples were incubated at 37°C for 45 min. Following this, iodoacetamide was added to the sample at a final concentration of 25 mmol/L and the sample was incubated at room temperature for 55 min in the dark. Next, the sample was diluted with 100 mmol/L triethylammonium carbonate (TEAB) buffer until the urea concentration was <2 mol/L. Trypsin (Hualishi, China, #HLS TRY001C) was then added at a mass ratio of 1:50, and the sample mixture was digested overnight at 37°C. The next morning, trypsin was again added at a mass ratio of 1:100, and the sample mixture was further digested at 37°C for 4 h. Strata X C18 (Phenomenex) was used to desalt the protein sample after digestion, and the peptides were labeled according to the iTRAQ^®^ Reagent-8PLEX One Assay Kit (Sigma-Aldrich, #4381662). Briefly, peptides were dissolved in an appropriate volume of 100 mmol/L TEAB. Next, 41 μL of acetonitrile was reconstituted with 0.8 mg of iTRAQ reagent and combined with 100 μg of peptides for 1 h at room temperature, and hydroxylamine was added to terminate the labeling reaction. Next, the sample was vacuum dried. The yak samples were labeled with iTRAQ113, iTRAQ114, and iTRAQ116, while the cattle samples were labeled with iTRAQ118, iTRAQ119, and iTRAQ121. The labeled samples were then mixed and vacuum dried.

### LC-MS/MS Analysis and Mass Spectrometry Data Search

LC-MS/MS detection was performed on a hybrid quadrupole-time of flight LC-MS/MS mass spectrometer coupled with a nanospray source. Peptides were loaded onto a C18 trap column (Thermo Scientific, USA) and then eluted into a C18 analytical column (1.9 mm, 150 μm ×120mm, Thermo Scientific, USA). Three independent biological replicates were used for each sample, and peptides from these biological replicates were independently analyzed by the LC-MS/MS. Raw Thermo Scientific Q Exactive mass spectrometer was used to obtain the raw mass data in the RAW format. Thermo Proteome Discoverer 1.4 (Thermo Fisher, version 1.4.0.288) was used to convert the RAW file into the MGF format. ProteinPilot (software version: 4.5) was used to analyze the mass spectroscopy data. After exporting the data to Excel, DEPs were screened according to the criteria of standard false discovery rate (FDR) <0.01. Any proteins with a differential score >1.3, a peptide number ≥2, a difference >1.5 (upregulated) or <0.67 (downregulated), and a *P* < 0.05 were selected as a DAP. The mass spectrometry proteomics data were deposited to the ProteomeXchange Consortium via PRIDE (16) partner repository with the dataset identifier PXD024100.

### Bioinformatics Analysis

The bovine gene and protein databases were used as the alignment core. The DAPs identified in this study were cattle (*Bos taurus*) orthologous proteins. Protein information annotation, Gene Ontology (GO) analysis, and Reactome pathway analysis were performed using the ClusterProfiler package in R project [http://www.r-project.org; (17)], Uniprot protein database [https://www.uniprot.org, UniProt release 2019_11; (18)], and the Reactome database [https://reactome.org/; (19)]. Inter-protein interaction analysis was performed using STRING [https://string-db.org, version 11; (20)].

### Western Blot Analysis

To confirm the DAPs identified by iTRAQ, we randomly selected two DAPs (one upregulated and one downregulated). The primary antibodies used were rabbit anti-FKBP52 antibody (Beijing Biosynthesis Biotechnology, #bs-11270R) and anti-ATPAF2 antibody-middle region (Aviva Systems Biology, #ARP62162_P050) diluted to 1:1,200. The DAPs were probed with antibodies against glyceraldehyde-3-phosphate dehydrogenase (1: 3,000, Aviva Systems Biology, #OAAJ03302). The HRP-labeled anti-rabbit/mouse IgG (1:4,000, ProteinTech, #10285-1 AP) were used as secondary antibodies. Chemiluminescent detection was performed using Beyo ECL Star (Beyotime, #P0018,) and the ProteinSimple Multi-Function Imager was used for exposure, imaging, and densitometry. Western blotting was performed in triplicate and one-way analysis of variance (ANOVA) was performed using the R project.

## Results

### Identification of Differentially Abundant Proteins by iTRAQ

To determine DAPs in mitochondria between yak and cattle, we utilized a proteomics approach based on iTRAQ quantification. In total, 4,517 proteins were identified in the mitochondrial fraction samples, of which the global proteomic analysis detected a total of 370 proteins in yak and cattle brain tissue, each of which had at least two peptides identified with a minimum unused score of ≥1.3. This indicated a >95% confidence in correct sequence identification (Supplementary Table 1). Further analysis was performed on those proteins with a coefficient of variation <50%. Comparison of the mitochondrial proteomes identified 57 proteins that met analysis criteria: average fold change of ≥1.5 or ≤ 0.67, and *P* < 0.05, of which 35 proteins were upregulated and 22 were downregulated in yak compared with cattle (Supplementary Table 2). Four proteins were identified with a fold-change value lower than 0.5. These strongly downregulated proteins included short/branched chain specific acyl-CoA dehydrogenase (ACADSB), peptidylprolyl isomerase (FKBP4), phosphodiesterase (PDE2A), and pyrroline-5-carboxylate reductase 2 (PYCR2). The average fold-change of FKBP4 was the lowest (0.34). Eight proteins were identified with a fold-change value >2.0. These strongly upregulated proteins included aldehyde dehydrogenase (ALDH2), fumarate hydratase (FH), hydroxyacyl-CoA dehydrogenase (HADH), nucleolar protein 3 (NOL3), oxoglutarate dehydrogenase-like (OGDHL), cytochrome c oxidase subunit NDUFA4 (NDUFA4), sirtuin 2 (SIRT2), and isocitrate dehydrogenase (NADP) (IDH1). Among these, ALDH2 and NOL3 were the most upregulated, with fold-change values of 3.05 and 2.77, respectively.

### GO Analysis of Mitochondrial DAPs

**Figure 2:**
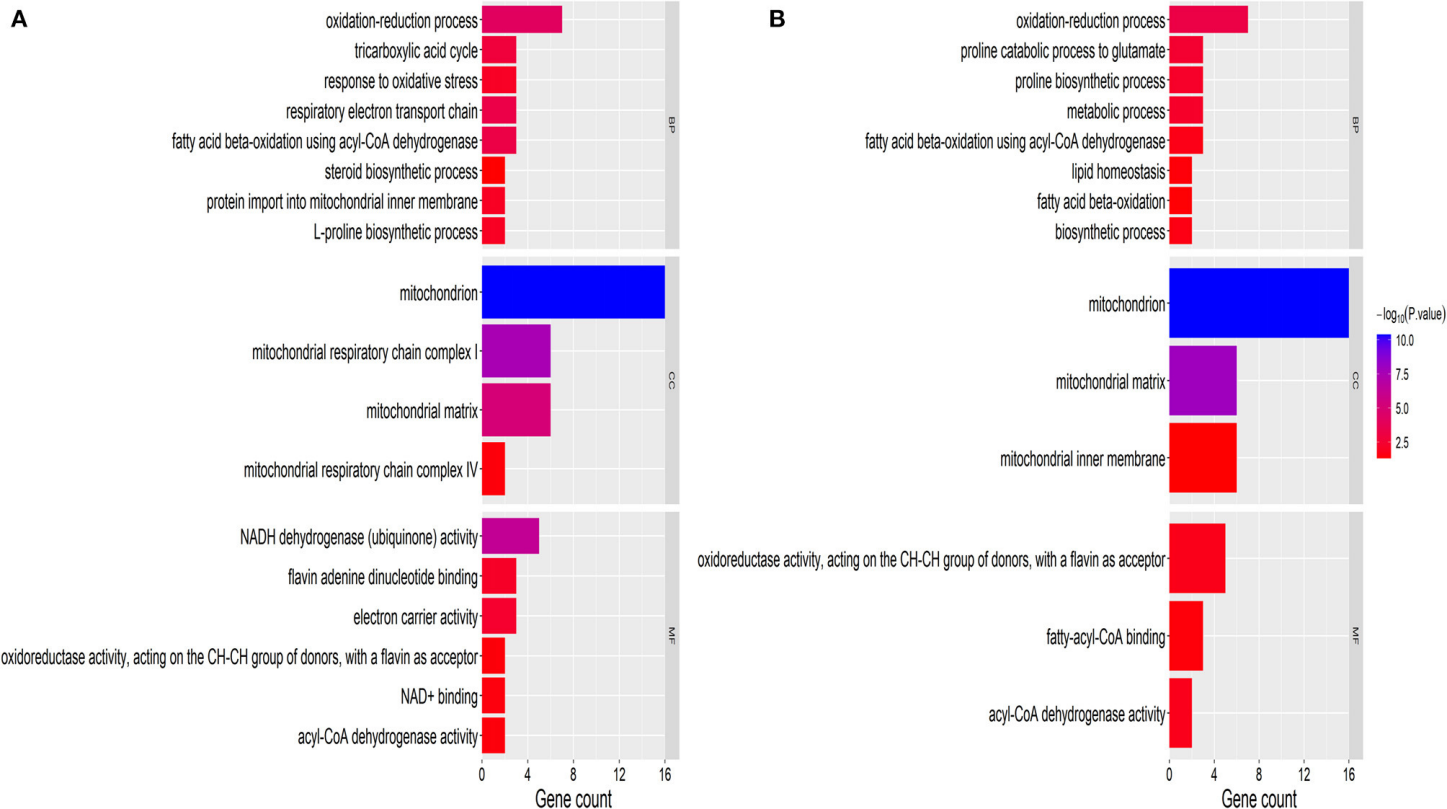
GO classification of DAPs. **(A)** Upregulated proteins. **(B)** Downregulated proteins. The x-axis represents GO terms. The y-axis represents the number of enriched proteins within each primary category. MF, molecular function; BP, biological process; and CC, cellular component.

The GO definition was used to describe the functional concept of the gene and the relationship between DAPs (yak and cattle), including MF (molecular function), BP (biological process), and CC (cellular component) (21). To identify GO functional groups enriched in DAPs, the GO database and genomic annotation information of cattle were used in the GO analyses. The 18 upregulated DAPs were significantly enriched (*P* < 0.01) for GO terms, including 6 MF terms, 8 BP terms, and 4 CC terms (Figure 2A and Supplementary Table 3). The 14 downregulated DAPs were significantly enriched (*P* < 0.01) for GO terms, including 3 MF terms, 8 BP terms, and 3 CC terms (Figure 2B). Among the upregulated DAPs, the enriched MF GO terms indicated that DAPs were mainly associated with NADH dehydrogenase (ubiquinone) activity, flavin adenine dinucleotide binding, and electron carrier activity. The top two GO terms were mitochondrion and mitochondrial matrix. Among the downregulated DAPs, the enriched GO terms included mitochondrion, oxidation–reduction process, and metabolic process.

### Pathway Enrichment Analysis of Mitochondrial DAPs

**Figure 3:**
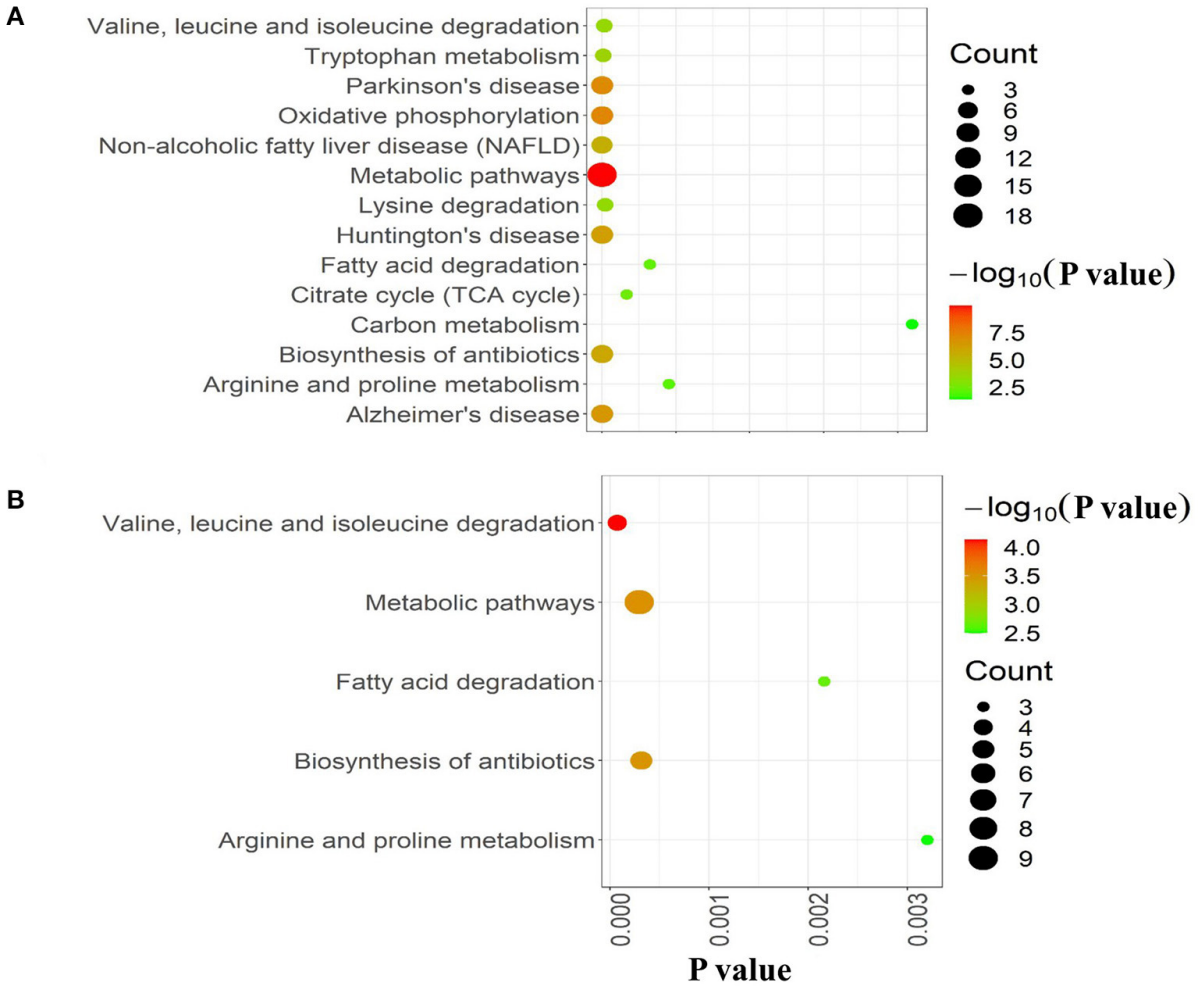
Reactome pathway analysis of DAPs. **(A)** Upregulated and **(B)** downregulated proteins in the mitochondria of yak brain tissue compared with that of cattle. The x-axis shows each of the enriched pathways. The y-axis represents the number of differentially abundant proteins (DAPs) in each primary category.

To further explore the molecular mechanism underlying the plateau adaptability in yaks, we used the Reactome pathway database to assess the enrichment of metabolic pathways in the DAPs (yak vs. cattle). The enrichment results indicated that the 35 upregulated DAPs were involved in 12 pathways (FDR <0.05), including citric acid (TCA) cycle, respiratory electron transport, and pyruvate metabolism (Figure 3A and Table 1). Twenty-one downregulated DAPs included seven pathways (FDR <0.05), including metabolism, branched-chain amino acid catabolism, and mitochondrial beta-oxidation of unsaturated fatty acids (Figure 3B and Table 1). Therefore, the pathway analysis showed that the majority of the upregulated DAPs were related to the TCA cycle and respiratory electron transport, whereas the downregulated DAPs were related to mitochondrial fatty acid beta-oxidation and metabolism of amino acids and derivatives.

**Table 1:** Key pathways related to brain mitochondrial metabolism.

**Pathways**	**FDR**	**DAPs**
The citric acid (TCA) cycle and respiratory electron transport (up)	2.97E10-11	NDUFA8, FH, NDUFA5, NDUFB5, NDUFA4, COX4I1, IDH1, ETFDH, ETFA, NDUFS8, NDUFAB1, ATP5D, D2HGDH
Respiratory electron transport (up)	4.06E10-9	NDUFA8, NDUFS8, NDUFA5, NDUFB5, NDUFA4, COX4I1, NDUFAB1, ETFDH, ETFA
Pyruvate metabolism and Citric TCA cycle (up)	0.031	FH, IDH1, D2HGDH
Metabolism of amino acids and derivatives (down)	4.98E10-5	ALDH4A1, GSTZ1, AADAT, MCCC1, BCKDHB, PYCR2, PRODH, ACADSB
Branched-chain amino acid catabolism (down)	0.002	MCCC1, BCKDHB, ACADSB
mitochondrial fatty acid beta-oxidation of unsaturated fatty acids (down)	0.006	ECI1, ACADM

### Functional Protein Association Networks of Mitochondrial DAPs

**Figure 4:**
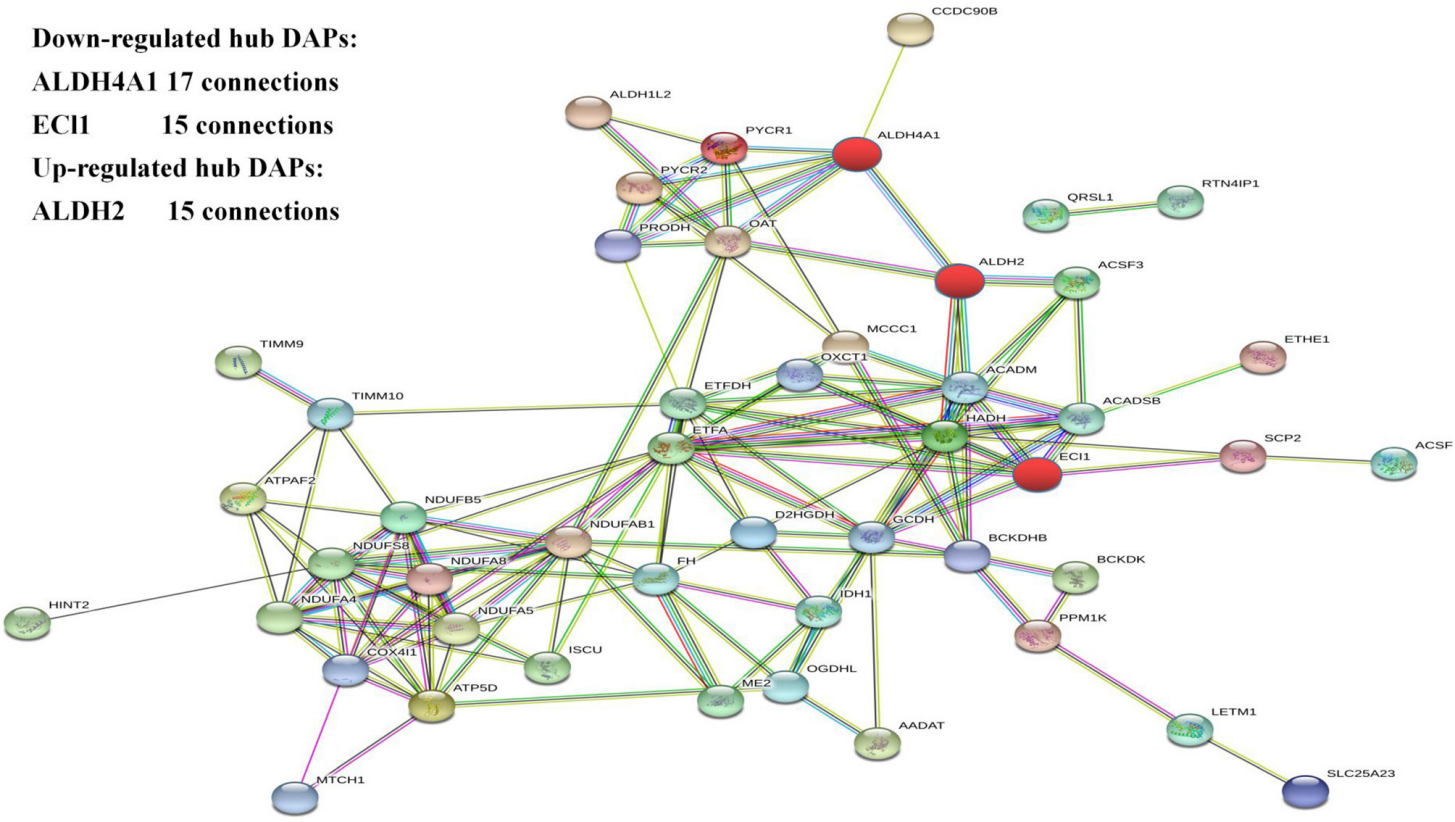
The protein–protein interaction network generated and visualized using STRING.

The protein–protein interaction network of the DAPs identified in this study was analyzed using the Search Tool for the Retrieval of Interacting Proteins 11 (STRING 11) database. After removing unconnected proteins and self-loops, the PPI network identified protein nodes (Figure 4). Proteins with higher connections than others in the network were designated key hubs, which may play significant roles in the network adjustment. Association analysis showed the top three key hub proteins to be ALDH4A1 (17 nodes), ALDH2 (15 nodes), and ECI1 (15 nodes).

### Verification and Analysis of Western Blots

**Figure 5:**
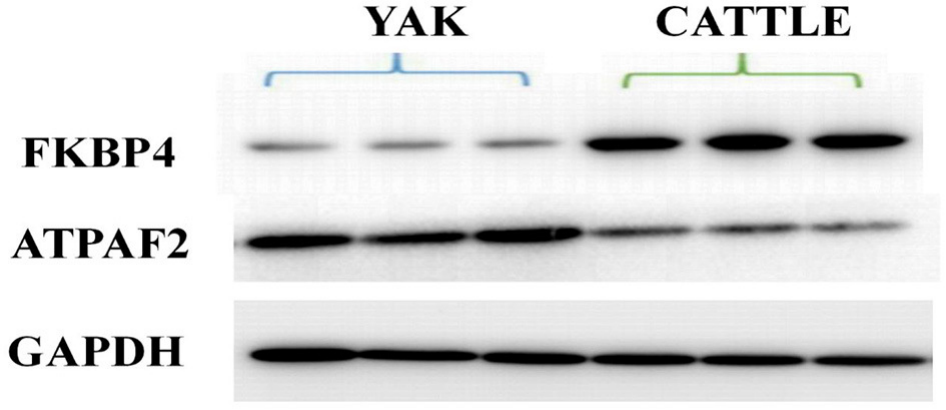
Western blot of FKBP4 and ATPAF2.

Western blot analysis was performed to verify the results from iTRAQ proteomics. Immunoblotting demonstrated that the expression of peptidylprolyl isomerase (FKBP4) in mitochondria from the brain tissue of yak was significantly lower than that found in cattle (*P* < 0.01; Figure 5). The average ratio of FKBP4 expression in yak compared to that in cattle from western blotting was ~0.3026, which was slightly lower than the ratio 0.3433 from iTRAQ (Supplementary Table 4). In contrast, the expression of ATP synthase mitochondrial F1 complex assembly factor 2 (ATPAF2) from the brain tissue of yak was significantly higher than that found in cattle (*P* < 0.01; Figure 5). The average ratio of ATPAF2 expression in yak compared to that in cattle from western blotting was ~1.2786 (Supplementary Table 4), which was comparable to the ratio of 1.5034 from iTRAQ. Altogether, these data suggested that the immunoblotting results verified the results obtained from iTRAQ proteomics.

## Discussion

Yak is a unique livestock species on the QTP, and its adaptation to harsh alpine conditions, including low oxygen partial pressure, high ultraviolet radiations, low grass quality, is much higher than that of its close relatives dwelling at low altitudes (22). For grazing yaks, the efficient use of nutrients and energy is essential for survival through the long winter (23). Previous studies have shown that the yak trachea, lungs, heart, and other organs are structurally adapted to plateau conditions (24). Compared with their close relatives such as cattle, these changes include shortening of the trachea, increased thickness of the alveolar septum, thickening of the smooth muscle, and a higher blood cell density (25). These structural adaptations bestow the yak with a high blood oxygen transport capacity and mitochondrial density, allowing efficient aerobic metabolism.

Brain tissue, including the brain, cerebellum, and brainstem, is the highest energy-consuming tissue, regulating homeostatic body functions through neural activity. Considering the important functions of mitochondria, we sought to identify mitochondrial proteins that were significantly differentially expressed DAPs between yak and cattle, and conducted bioinformatics analysis on the identified proteins. Such DAPs may provide molecular insights into yak survival in the harsh plateau environment.

Bioinformatics analysis of downregulated DAPs showed them to be implicated in several key pathways, such as metabolism of amino acids and their derivatives, as well as metabolism of branched amino acids (Table 1). Valine, leucine, and isoleucine are branched-chain amino acids (BCAAs) that are important for protein synthesis and brain activity (26). BCAAs, which are believed to enter the brain through the bloodstream and reduce the brain's production of serotonin, cause fatigue and reduced mental fatigue by decreasing serotonin levels (27). Recent studies have shown that glutamate is available to both neurons, and astrocytes, and that when glutamate is present, astrocytes use glucose less, and ATP levels in astrocytes are higher when glucose is administered. The blood–brain barrier blocks the flow of glutamate into the brain; therefore, glutamate has little role in supporting brain energy production (28). The upregulated DAP pathways suggested that the TCA cycle and respiratory electron transport are the most significant pathways (2.97E-11). A further molecular adaptation may be associated with the TCA cycle and respiratory electron transport. The TCA cycle is the converging metabolic pathway for the catabolism of three major nutrients (sugars, lipids, and amino acids) to release energy. As part of the oxidative breakdown of carbohydrates, FH hydrates fumarate to (S)-malate in the TCA cycle. Malic acid, the product of the TCA cycle, plays a key role in the downstream electron transfer and ATP synthesis (29, 30). At low concentrations, ATP activates the heterotetramers and the heterodimer composed of IDH3A and IDH3G subunits; however, it inhibits their activities at high concentrations. In addition, ATP exhibits only an inhibitory effect on the heterodimer composed of IDH3A and IDH3B subunits (31). In addition, malic enzyme (ME2) plays a significant role in the TCA pathway. It is involved in the de-acidification of malic acid, NAD binding, and metal ion binding, which could produce ATP to power cellular metabolic activity (32, 33). Another potential source of molecular adaptation may be associated with the biogenesis of complex I. NADH dehydrogenase (NDUF) is a large protein comprising several protein sub-complexes (34). Complex I, which is a secondary subunit of the mitochondrial membrane respiratory chain NADH dehydrogenase, does not participate in catalysis (35). Complex I is involved in electron transfer from NADH to the respiratory chain. The direct electron acceptor of this enzyme is ubiquinone. Enhanced biogenesis of this complex may promote energy release through the electron transport chain. Overall, the upregulated DAPs suggest that yaks may have a highly efficient TCA cycle and energy utilization mechanism, allowing them to survive a long winter with nutrient shortage.

The most enriched hub proteins were found to be ALDH4A1, ALDH2, and ECI1. ALDH is widely involved in the oxidation of aldehydes in the body. Excess aldehydes accumulate in the tissues and organs causing irreversible damage. ALDH4A1 and ALDH2 belong to the aldehyde dehydrogenase family. ALDH2 is located in the mitochondria and widely present in the human heart, brain, lungs, kidneys, liver, and other tissues. The *ALDH2* gene in the ALDH2 acetaldehyde dehydrogenase family is the only one with high transcription in the brain (36). The dehydrogenase activity of ALDH2 converts acetaldehydes to acetic acid, thereby reducing their cytotoxicity and protecting tissues and organs. Increasing the activity of ALDH2 protects again neurological damage (37). In addition, hypoxia can increase lipid peroxidation causing increased lipid peroxidation products, including 4-HNE (4-hydroxynonenal) through the LO (lipoxygenase) pathway and ROS (reactive oxygen species) pathway. Studies have found that ALDH2 regulates mitochondrial division through the 4-hydroxyenoic acid/HIF (hypoxia inducible factor)-1α/Drp1 (motility-related protein 1) signaling pathway (37). ALDH4A1 can encode a pyrroline-5-carboxylic acid dehydrogenase involved in the degradation of proline, which catalyzes the conversion of pyrroline-5-carboxylic acid to glutamic acid (38). ALDH4A1 deficiency leads to hyperprolinemia; patients present with epilepsy and intellectual disability (39, 40). These proteins are mainly involved in bioenergy metabolism in hypoxic environments, suggesting that they may contribute to hypoxic adaptation mechanisms.

## Conclusions

In summary, this is the first study to provide proteomic data to support a molecular theory of plateau adaptability in yaks. We identified key DAPs; the majority of the upregulated DAPs were related to the TCA cycle and respiratory electron transport, whereas the downregulated DAPs were related to mitochondrial fatty acid beta-oxidation and metabolism of amino acids and derivatives. Hypoxia at high altitudes can regulate the activity of the mitochondrial respiratory chain. Changes in the mitochondrial structure and function are one of the important mechanisms in animals to overcome the hypoxic environment. Under a low oxygen environment, yak's endurance is enhanced as compared to that of cattle. We believe that these results will enhance our understanding of the underlying genetic and molecular mechanisms promoting plateau adaptability of yaks.

## Data Availability Statement

The datasets presented in this study can be found in online repositories. The names of the repository/repositories and accession number(s) can be found at: iProX; IPX0002625000.

## Ethics Statement

The animal study was reviewed and approved by all yaks and cattle involved in this study were in compliance with the guidelines for the care and use of laboratory animals issued by the State Council of the People's Republic of China. Additionally, the current investigation was approved by the Animal Management and Ethics Committee of the Lanzhou Institute of Animal Science and Veterinary Medicine, Chinese Academy of Agricultural Sciences (Permit No. SYXK-2016-0039).

## Author Contributions

PY and CL: conceptualization, project administration, resources, and supervision. XM and QZ: data curation, validation, and visualization. XM, QZ, and CL: formal analysis. XM and PY: investigation. DF, YL, HJ, PB, XW, and MC: methodology. XM: software and writing—original draft. XM, QZ, DF, HJ, PB, XW, MC, and XG: writing—review and editing. All authors contributed to the article and approved the, submitted version.

## Conflict of Interest

The authors declare that the research was conducted in the absence of any commercial or financial relationships that could be construed as a potential conflict of interest.

## Publisher's Note

All claims expressed in this article are solely those of the authors and do not necessarily represent those of their affiliated organizations, or those of the publisher, the editors and the reviewers. Any product that may be evaluated in this article, or claim that may be made by its manufacturer, is not guaranteed or endorsed by the publisher.

## References

[1] Qiu Q Zhang G Ma T Qian W Wang J Ye Z. The yak genome and adaptation to life at high altitude. Nat Genet. (2012) 44:946–9. 10.1038/ng.2343 22751099

[2] Guo X Yan P Liang CN Pei J. Developmental situations and countermeasures of yak industry in China. China Cattle Sci. (2009) 35:55–7.

[3] Ding L Wang Y Brosh A Chen J Gibb M Shang Z. Seasonal heat production and energy balance of grazing yaks on the Qinghai-Tibetan plateau. Anim Feed Sci Technol. (2014) 198:83–93. 10.1016/j.anifeedsci.2014.09.022

[4] Guo S Savolainen P Su J Qian Z Qi D Jie Z. Origin of mitochondrial DNA diversity of domestic yaks. BMC Evol Biol. (2006) 6:73–73. 10.1186/1471-2148-6-73 16995938 PMC1626082

[5] Lai S-J Wang L Liu Y-P Li X-W. Study on mitochondrial DNA genetic polymorphism of some yak breeds in China. Acta genet Sin. (2005) 32:463–70. 10.3321/j.issn:0366-6964.2005.09.006 16018255

[6] Raichle M Gusnard D. Appraising the brain's energy budget. Proc Natl Acad Sci USA. (2002) 99:10237–9. 10.1073/pnas.172399499 12149485 PMC124895

[7] Xing C Tarumi T Liu J Zhang Y Turner M Riley J. Distribution of cardiac output to the brain across the adult lifespan. J Cereb Blood Flow Metab. (2016) 37:2848–56. 10.1177/0271678X16676826 27789785 PMC5536794

[8] Attwell D Laughlin S. An energy budget for signaling in the grey matter of the brain. J Cereb Blood Flow Metab. (2001) 21:1133–45. 10.1097/00004647-200110000-00001 11598490

[9] Hyder F Fulbright R Shulman R Rothman D. Glutamatergic function in the resting awake human brain is supported by uniformly high oxidative energy. J Cereb Blood Flow Metab. (2013) 33:339–47. 10.1038/jcbfm.2012.207 23299240 PMC3587823

[10] Shao B-P Ding Y-P Xie Z Yu H-X Brand-Saberi B Wang J. The cranial cervical ganglion and its branches in the yak (*Bos grunniens*). Vet J. (2007) 173:174–7. 10.1016/j.tvjl.2005.08.016 16246603

[11] Shao B-P Ding Y-P Yu S-Y Wang J. The arterial supply of the eye of the yak (*Bos grunniens*). Res Vet Sci. (2008) 84:174–7. 10.1016/j.rvsc.2007.04.019 17950766

[12] Long L Zhu Y Li Z Zhang H Liu L Bai J. Differential expression of skeletal muscle mitochondrial proteins in yak, dzo, and cattle: a proteomics-based study. J Vet Med Sci. (2020) 82:1178–86. 10.1292/jvms.19-0218 32641622 PMC7468061

[13] Xin JW Chai ZX Zhang CF Zhang Q Zhu Y Cao HW. Signature of high altitude adaptation in the gluteus proteome of the yak. J Exp Zool B Mol Dev Evol. (2020) 334:362–72. 10.1002/jez.b.22995 32779369

[14] Ballard JW Whitlock MC. The incomplete natural history of mitochondria. Mol Ecol. (2004) 13:729–44. 10.1046/j.1365-294x.2003.02063.x 15012752

[15] Balaban RS Nemoto S Finkel T. Mitochondria, oxidants, and aging. Cell. (2005) 120:483–95. 10.1016/j.cell.2005.02.001 15734681

[16] Perez-Riverol Y Csordas A Bai J Bernal-Llinares M Hewapathirana S Kundu DJ. The PRIDE database and related tools and resources in 2019: improving support for quantification data. Nucleic Acids Res. (2019) 47:D442–50. 10.1093/nar/gky1106 30395289 PMC6323896

[17] Yu G Wang LG Han Y He QY. clusterProfiler: an R package for comparing biological themes among gene clusters. OMICS. (2012) 16:284–7. 10.1089/omi.2011.0118 22455463 PMC3339379

[18] Gane P Bateman A Mj M O'donovan C Magrane M Apweiler R. UniProt: a hub for protein information. Nucleic Acids Res. (2015) 43:D204–212. 10.1093/nar/gku989 25348405 PMC4384041

[19] Jassal B Matthews L Viteri G Gong C Lorente P Fabregat A. The reactome pathway knowledgebase. Nucleic Acids Res. (2020) 48:D498–503. 10.1093/nar/gkz1031 31691815 PMC7145712

[20] Szklarczyk D Gable AL Lyon D Junge A Wyder S Huerta-Cepas J. STRING v11: protein-protein association networks with increased coverage, supporting functional discovery in genome-wide experimental datasets. Nucleic Acids Res. (2019) 47:D607–13. 10.1093/nar/gky1131 30476243 PMC6323986

[21] Camon E Magrane M Barrell D Lee V Dimmer E Maslen J. The Gene Ontology Annotation (GOA) database: sharing knowledge in Uniprot with Gene Ontology. Nucleic Acids Res. (2004) 32:D262–6. 10.1093/nar/gkh021 14681408 PMC308756

[22] Lalthantluanga R Wiesner H Braunitzer G. Studies on yak hemoglobin (*Bos grunniens*, Bovidae): structural basis for high intrinsic oxygen affinity? Biol Chem Hoppe Seyler. (1985) 366:63–8. 10.1515/bchm3.1985.366.1.63 4005038

[23] Deng F Xia C Jia X Song T Liu J Lai SJ. Comparative study on the genetic diversity of *GHR* gene in Tibetan cattle and Holstein cows. Anim Biotechnol. (2015) 26:217–21. 10.1080/10495398.2014.993082 25927168

[24] Durmowicz AG Hofmeister S Kadyraliev TK Aldashev AA Stenmark KR. Functional and structural adaptation of the yak pulmonary circulation to residence at high altitude. J Appl Physiol. (1993) 74:2276–85. 10.1152/jappl.1993.74.5.2276 8335557

[25] Betkier-Lipińska K Suwalski G Czarkowski S Hendzel P Cwetsch A. Pulmonary artery aneurysm in an adult patient with idiopathic dilatation of the pulmonary artery. Kardiochir Torakochirurgia Pol. (2015) 12:341–4. 10.5114/kitp.2015.56785 26855651 PMC4735536

[26] Sperringer JE Addington A Hutson SM. Branched-chain amino acids and brain metabolism. Neurochem Res. (2017) 42:1697–709. 10.1007/s11064-017-2261-5 28417264

[27] Asor E Stempler S Avital A Klein E Ruppin E Ben-Shachar D. The role of branched chain amino acid and tryptophan metabolism in rat's behavioral diversity: Intertwined peripheral and brain effects. Eur Neuropsychopharmacol. (2015) 25:1695–705. 10.1016/j.euroneuro.2015.07.009 26271721

[28] Chaves C Remiao F Cisternino S Decleves X. Opioids and the blood-brain barrier: A bynamic interaction with consequences on drug disposition in brain. Curr Neuropharmacol. (2017) 15:1156–73. 10.2174/1570159x15666170504095823 28474563 PMC5725546

[29] King A Selak MA Gottlieb E. Succinate dehydrogenase and fumarate hydratase: linking mitochondrial dysfunction and cancer. Oncogene. (2006) 25:4675–82. 10.1038/sj.onc.1209594 16892081

[30] Lehtonen HJ Kiuru M Ylisaukko-Oja SK Salovaara R Herva R Koivisto PA. Increased risk of cancer in patients with fumarate hydratase germline mutation. J Med Genet. (2006) 43:523–6. 10.1136/jmg.2005.036400 16155190 PMC2564537

[31] Balss J Meyer J Mueller W Korshunov A Hartmann C Von Deimling A. Analysis of the *IDH1* codon 132 mutation in brain tumors. Acta Neuropathol. (2008) 116:597–602. 10.1007/s00401-008-0455-2 18985363

[32] Castelein H Gulick T Declercq PE Mannaerts GP Moore DD Baes MI. The peroxisome proliferator activated receptor regulates malic enzyme gene expression. J Biol Chem. (1994) 269:26754–8. 10.1016/S0021-9258(18)47083-4 7929410

[33] Jiang P Du W Mancuso A Wellen KE Yang X. Reciprocal regulation of p53 and malic enzymes modulates metabolism and senescence. Nature. (2013) 493:689–93. 10.1038/nature11776 23334421 PMC3561500

[34] Weiss H Friedrich T Hofhaus G Preis D. The respiratory-chain NADH dehydrogenase (complex I) of mitochondria. Eur J Biochem. (1991) 197:563–76. 10.1111/j.1432-1033.1991.tb15945.x 2029890

[35] Kocher TD Conroy JA Mckaye KR Stauffer JR Lockwood SF. Evolution of NADH dehydrogenase subunit 2 in east African cichlid fish. Mol Phylogenet Evol. (1995) 4:420–32. 10.1006/mpev.1995.1039 8747298

[36] Chen C Budas G Churchill E Disatnik M-H Hurley T Mochly-Rosen D. Activation of aldehyde dehydrogenase-2 reduces ischemic damage to the heart. Science. (2008) 321:1493–5. 10.1126/science.1158554 18787169 PMC2741612

[37] Zhao Y Wang B Zhang J He D Zhang Q Pan C. ALDH2 (aldehyde dehydrogenase 2) protects against hypoxia-induced pulmonary hypertension. Arterioscler Thromb Vasc Biol. (2019) 39:2303–19. 10.1161/ATVBAHA.119.312946 31510791

[38] Marchitti S Brocker C Stagos D Vasiliou V. Non-P450 aldehyde oxidizing enzymes: the aldehyde dehydrogenase superfamily. Expert Opin Drug Metab Toxicol. (2008) 4:697–720. 10.1517/17425255.4.6.697 18611112 PMC2658643

[39] Srivastava D Singh R Moxley M Henzl M Becker D Tanner J. The three-dimensional sstructural basis of type II hyperprolinemia. J Mol Biol. (2012) 420:176–89. 10.1016/j.jmb.2012.04.010 22516612 PMC3372638

[40] Mustafavi S Samee A Siddiqui S Yousra T. A case report on achalasia cardia type - II. Int J Surg. (2019) 6:3401–5. 10.18203/2349-2902.isj20194086

